# Responses of Trace Elements to Aerobic Maximal Exercise in Elite Sportsmen

**DOI:** 10.5539/gjhs.v6n3p90

**Published:** 2014-02-21

**Authors:** Aynur OTAĞ, Muhsin HAZAR, İlhan OTAĞ, Alper Cenk Gürkan, İlyas Okan

**Affiliations:** 1 Cumhuriyet Üniversity, School of Physical Education and Sports, Sivas, Turkey; 2 Gazi Üniversity, School of Physical Education and Sports, Ankara, Turkey; 3 Cumhuriyet Üniversity, Vocational School of Health Services, Sivas, Turkey

**Keywords:** elite athlete, exercise, aerobic exercise, metabolism, performance

## Abstract

Trace elements are chemical elements needed in minute quantities for the proper growth, development, and physiology of the organism. In biochemistry, a trace element is also referred to as a micronutrient. Trace elements, such as nickel, cadmium, aluminum, silver, chromium, molybdenum, germanium, tin, titanium, tungsten, scandium, are found naturally in the environment and human exposure derives from a variety of sources, including air, drinking water and food. The Purpose of this study was investigated the effect of aerobic maximal intensity endurance exercise on serum trace elements as well-trained individuals of 28 wrestlers (age (year) 19.64±1.13, weight (Kg) 70.07 ± 15.69, height (cm) 176.97 ± 6.69) during and after a 2000 meter Ergometer test protocol was used to perform aerobic (75 %) maximal endurance exercise. Trace element serum levels were analyzed from blood samples taken before, immediately after and one hour after the exercise. While an increase was detected in Chromium (Cr), Nickel (Ni), Molybdenum (Mo) and Titanium (Ti) serum levels immediately after the exercise, a decrease was detected in Aluminum (Al), Scandium (Sc) and Tungsten (W) serum levels. Except for aluminum, the trace elements we worked on showed statistically meaningful responses (P<0.05 and P<0.001). According to the responses of trace elements to the exercise showed us the selection and application of the convenient sport is important not only in terms of sportsman performance but also in terms of future healthy life plans and clinically.

## 1. Introduction

98% of human body weight is composed of mainly six elements which are oxygen (O), carbon (C), hydrogen (H), nitrogen (N), calcium (CA), phosphorus (P) and additionally sulfur (S), potassium (K), sodium (Na), chlorine (Cl), magnesium (Mg) and silicon (Si). Except for inert gases having less possibility to have physiologic functions, 71 elements are called as trace elements due to their presence in living cells in small amounts (0.01-100tmg/kg). As trace elements perform an enzyme component in biologic systems or as catalyst in chemical reactions occurring within the cells, and as they have toxic effects, it is known that insufficient or excessive intake of these elements causes many diseases (Aras, 2006). Due to important qualities of trace elements, knowledge of the responses given by serum levels to the exercise types will be useful for clinics, selection of exercise type and also for the selection of treatment program to be applied (Haroa, 2011).

Trace elements are found inherently in the environment and people are subject to these elements through various resources such as air, drinking water and foods. World Health Organization defined 19 trace elements which are important for human health (Parkin, 2005). The trace elements in the human body can be classified as those proved to be useful, those whose benefit is still unknown and possibly toxic elements. Chromium, iron, cobalt, copper, zinc, selenium, molybdenum and iodine are useful trace elements and they should be taken in small amounts for continuance of life. Lack of these elements may result in important disorders in the body and even in death. While manganese, silicon, nickel, boron, vanadium and tin are possibly useful trace elements; fluorine, arsenic, cadmium, lead, aluminum and mercury are possibly toxic elements (Aras, 2006).

Measurement capabilities of the methods used to measure trace elements in biology and environmental samples are depending on specimen (blood, urine, hair, nail) and type of preparing the specimens for analysis (Meyer F, 1987). In order to meet the increasing requirements of today, automatic analysis methods were developed which are used in the detection of trace elements. These methods, providing data numerously and fast with little contribution of the user are: Atomic Absorption Spectrometry (AAS), Electrothermal Absorption Spectrometry (ETAAS), Inductive Coupled Plasma Optical Emission Spectrometry (ICPOES), Inductive Coupled Plasma Mass Spectrometry (ICPMS), Atomic Fluoroscence Spectrometry (AFS), Neutron Activation Analysis (NAA), X-Ray Fluoroscence Spectroscopy (XRF). The most convenient methods for solution samples are AAS, ICP-OES and ICP-MS (Aras, 2006).

Chromium is a trace element which affects insulin activity positively and which is necessary for sucrose and fat metabolisms. It is found throughout the body. However, it is mostly found in liver, kidney, spleen and bone (Mertz, 1998; Vincent, 2003; Jacquamet, 2003). Although 30-60 mg chromium is taken daily, only 4-6 mg can be stored in the body (Kumpulainen, 1992). Molybdenum, one of the useful trace elements, performs at the active centers of some enzymes containing Flavin (Chan, 1998). Furthermore, molybdenum component enzymes are important also ecologically. They help carbon, nitrogen and sulfur circle significantly (Frausta DA Silva, 2001). Nickel is defined as s possibly useful trace element by the World Health Organization (Parkin, 2005). There are proofs showing that nickel is related to intracellular communication mechanism inhibition, fibroblast and epithelial cell eternity, DNA abnormalities and deletion induction, DNA, protein cross link construction, oxidative damage, nucleotide excision repair inhibition, and gene expression inactivation mechanisms in human beings (Miki, 1987; DI Paolo, 1979; Biederman, 1987; Patierno, 1993; Costa, 1996; Sen, 1987; Kosprzak, 1991; Hartwig, 1994; Lee, 1995). Aluminum is one of the most abundant in the world and possibly toxic trace elements (Aras, 2006). Although it wasn’t proven that aluminum has an important function in human beings and animals, it was pointed out that it affects some qualities such as growth, reproduction and milk production in some animal trials (Anke, 1990). The basic relation of aluminum element with human health is that it shows important toxic quality when it is found in big amounts (Alfrey, 1986). Scandium, titanium and tungsten are the trace elements of which effects on the organism are still not known (Aras, 2006). Scandium is a very rare element (Clayton, 2003). Titanium is an element which is not toxic, not rejected by the body, and which is used in surgical tools and implants (Emsley, 2001). Tungsten is an element which is widely used for industrial purposes.

In this study, we examined the responses given to aerobic maximal intensity endurance exercise by chromium, molybdenum and nickel among useful trace elements; aluminum among possibly toxic trace elements and scandium, titanium and tungsten among those whose benefit is still not proven in elite sportsmen of the wrestling team. We believe that analysis of the responses of trace elements to exercise will provide useful information in terms of both the health of sportsmen, sedentary with public health and healthy sporting planning.

## 2. Material and Method

Twenty-eight male subjects who are the elite wrestlers participated in this study. Experimental results on this subject were presented separately as a case report in the result section. The median age of the participating subjects was (year) ranging from 18 to 22. The other physical characteristics of the subjects were as follows table 1.

**Table 1:** Physical characteristic of the subjects

Age (year)	Weight (kg)	Height (cm)	BMI (kg/cm2)
19.64±1.13	70.07±15.69	176.97±6.69	22.37±1.22

The experimental protocol in this study was approved by the local ethics committee at Gazi University, Ankara, Turkey. All subjects were informed about the purpose and risks of the study before signing a written consent. Studies were performed according to the Declaration of Helsinki.

### 2.1 Exercise Protocol

A 2000 meter Ergometer test protocol was used to perform aerobic (75 %) maximal endurance exercise. Exercise tests were performed on a Concept IIC rowing Ergometer (Morrisville, USA). Subjects completed a 10 min warm-up before the exercise. All subjects were asked to cover a distance of 2000 m in the least time possible. The test was performed at ambient temperature (21 ± 0.5 ºC).

### 2.2 Blood Sampling

Blood samples were drawn from the antecubital vein of the subjects right before, immediately after, and one hour after exercise. Blood samples were collected in vacutainer tubes (Becton Dickinson, Franklin Lakes, NJ, USA) and centrifuged at 1500 g for 15 min. Serum samples were aliquoted and stored at -80 ºC until use for analyzing by inductively coupled plasma optical emission spectrometry (ICP-OES). Samples were only thawed once.

### 2.3 Sample Preparations and Measurements

On the 1 ml blood samples was added 2 ml HNO3 and the samples were digested in Berghof/Microwave Digestion system MWS-3 microwave apparatus. The microwave was kept at 160 °C for 5 min and at 190, 100 and 80 °C for 10 min each. The totally digested samples were diluted to 10 ml with the addition of deionized water 18.3 ohm cm-1. On the 1 ml blood samples was added 2 ml HNO3 and the samples were digested in Berghof/Microwave Digestion system MWS-3 microwave apparatus. The microwave was kept at 160 °C for 5 min and at 190, 100 and 80 °C for 10 min each. The totally digested samples were diluted to 10 ml with the addition of deionized water 18.3 ohm cm-1. Samples were analyzed directly using inductively coupled plasma optical emission spectrometry (ICP-OES, Perkin- Elmer, Optima 5300 DV, USA).

### 2.4 Statistical Analysis

Statistical analysis was performed with SPSS Ver. 16.0 for Windows. Statistical significance was set at p<0.05 (with 95% confidence levels). “Non-parametric Test’’ was used in the description of athletes data who included to study taking into the consideration expansiveness of the group. Wilcoxon signed - rank test was used to test the alterations of measured parameters due to time (before exercise, after exercise and one hour after exercise). Significance was defined as a *P* value *<* 0.05 in all groups.

## 3. Results

Serum Cr, Ni, Mo, Sc, Ti, W and Al levels as before training (assay1), immediately after training (assay2) and one hour after training (assay3). Have shown in Table 2.

**Table 2:** Serum Cr, Ni, Mo, Sc, Ti, W and Al levels as before training (assay1), immediately after training (assay2) and one hour after training (assay3)

Elements	Assay1	Assay2	Assay3	P (1,2)	P (2,3)	P (1,3)
Cr (µg/L)	13,42±3,12	15,23±4,12	16,18±4,65	0,010	0,466	0,008
Ni (µg/L)	3,14±0,54	6,29±11,00	4,60±2,46	0,000	0,982	0,000
Mo (µg/L)	17,14±1,12	17,42±4,91	16,89±0,58	0,007	0,008	0,333
Sc (µg/L)	0,93±0,13	0,90±0,65	0,87±0,07	0,000	0,000	0,039
Tİ (µg/L)	12,38±1,88	17,99±11,21	12,71±2,37	0,000	0,001	0,466
W (µg/L)	123,05±34,30	122,98±26,44	112,25±20,21	0,290	0,004	0,524
AI (µg/L)	81,33±117,48	76,91±31,80	72,89±60,57	0,068	0,076	0,425

Serum chromium levels have progressively increased. While difference between assay1 and assay2 was significant, difference between assay2 and assay3 was nonsignificant. Difference between assay1 and assay3 was significant.

Serum nickel and titanium levels have significantly increased immediately after training. One hour after training serum nickel and titanium levels have non significantly decreased. For nickel and titanium values of assay3 were higher than values of assay1 and differences between values of assay1 and assay3 were significant.

Serum molybdenum level has significantly increased immediately after training. One hour after training serum molybdenum level has non significantly decreased. Assay1 was higher than assay3 and difference between assay1 and assay3 was nonsignificant.

The levels of serum scandium, tungsten and aluminium were progressively decreased during the experiment. While differences between three assays were significant for scandium, they were nonsignificant for aluminium. For serum tungsten levels while difference between assay2 and assay3 was significant, both difference between assay1 and assay2 and difference between assay1 and assay3 were nonsignificant.

**Figure 1:**
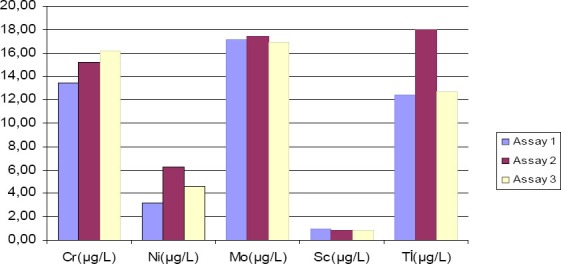
Serum Cr, Ni, Mo, Sc, Ti levels

**Figure 2:**
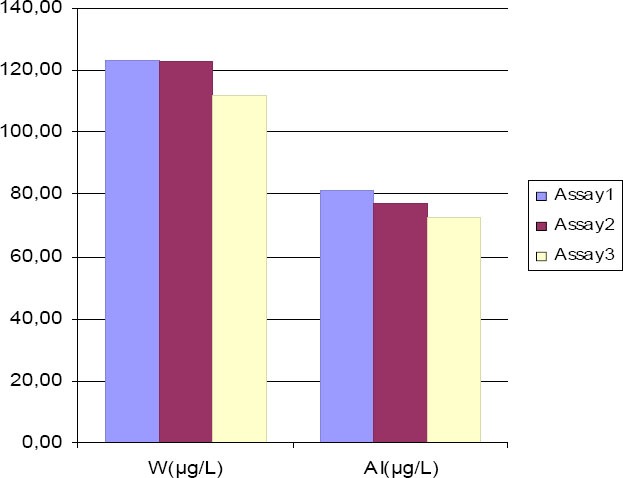
Serum W and Al levels

## 4. Discussion

Trace elements perform as a catalyst in chemical reactions occurring within the cells in biological systems or as an enzyme component (Aras, 2006). Due to these important functions, they also undertake important physiologic functions. In sportsmen, trace elements affect exercise performance with physiologic roles such as muscle contraction, normal cardiac rhythm, enzyme activation, neural response formation, oxidative phosphorescence, enzyme activation, oxygen transit, immune functions, acid-base balance, antioxidant activity and bone health. For this reason, sportsmen intake all these minerals in sufficient amounts for their diets in order to complete the normal process and increase their performance during the exercise (Speich, 2001; Hazar, 2012).

Knowledge of the responses given by trace elements to the exercise plays an important role in terms of health and performance of the sportsman as well as public health and exercise choice. In order to prevent mineral insufficiency, it should be analyzed which exercise type the trace elements give response and how they respond, and the appropriate diet should be determined under the light of this information (Kara, 2011; Baydil, 2013).

We researched the responses of chromium, nickel, molybdenum, scandium, titanium, tungsten and aluminum elements to aerobic maximal intensity endurance exercise we applied to elite sportsmen of the wrestling team.

Among trace elements, especially chromium affects muscle activity and lipid profiles positively and increases performance of the sportsman (Clarkson, 1997). Chromium may decrease cholesterol level (Efavi, 1993). It affects insulin activity positively (Vincent, 2003; Jacquament, 2003). While chromium deficiency increases blood glucose level, insulin, cholesterol and triglyceride level, it causes a decrease in body mass (Zafra-Stone, 2007). Furthermore, chromium deficiency contributes to some chronic diseases such as Type 2 diabetes and cardiovascular diseases (Preus, 1998). Immediately after aerobic maximal intensity endurance exercise we applied to elite sportsmen of the wrestling team, the serum level increased meaningfully and this increase continued even one hour later. Chromium increases during exercise will increase glucose and insulin activity and therefore use of glucose. Consequently, the exercise performance was contributed by producing more energy during the aerobic exercise. Moreover, the chromium, which increases during the exercise and then continues to increase later may contribute positively to body fat profile by decreasing cholesterol and triglyceride levels as well as contributing to the protection against Type 2 diabetes and cardiovascular diseases by contributing positively to insulin activity. In our study, while serum levels of nickel element increased meaningfully during the exercise, it decreased one hour after the exercise. In spite of this decrease, the serum level one hour after the exercise is meaningfully higher than the level before the exercise. Although marathon runners show a similar increase in nickel levels, this increase is meaningless (Berger, 2002). The difference between the responses of nickel serum level to exercise among sport branches may be related to the type of exercise. Nickel element affects glucose and insulin metabolism and iron usage positively (Kieffer, 1979). Thus, the meaningful increase of the nickel serum level during exercise ensures the glucose is included within the cell and used as a fuel for energy production. Furthermore, as the positive effect on iron usage will affect oxygen carriage positively, the increase in nickel serum level during the exercise may affect exercise performance positively. While molybdenum level increases during marathon run, it decreases, then (Berger, 2002). The fact that molybdenum serum level increases during the exercise and then decreases in our study shows that there are similar responses between the exercise applied and the marathon in terms of molybdenum. For important enzymes, molybdenum is necessary as a cofactor (Burtis, 2006). Increase of molybdenum during exercise is important for human health. However, being less of a molybdenum serum level after the exercise than the level before the exercise should be paid attention. Aluminum does not have any important physiological function (Anke, 1990). The most important relation of this element with the organism is that it shows toxicity when it is found in excessive amounts. Excessive aluminum affects the skeleton of decreasing bone formation noticeably and results in osteomalasy. Another pathologic result of aluminum toxicity is hypochromic anemia which is not related to iron deficiency (Alfrey, 1989). Moreover, aluminum may cause neurotoxicity in high doses by changing the function of blood-brain barrier (Banks, 1989). In our study, serum aluminum level decreased during the exercise (P>0.05) and this decrease continued even after the exercise, and it may have positive effects in terms of clinical and exercise performance. We detected meaningful changes in serum levels of scandium, titanium and tungsten elements during and after the exercise. These elements are those benefits to the organism of which are not determined yet (Aras, 2006). It would be advantageous to perform interdisciplinary studies in order to determine possible physiological roles of these elements and their responses to the exercise.

In our study, we researched the responses given to aerobic maximal intensity endurance exercise by chromium, nickel and molybdenum from useful trace elements; aluminum from possibly toxic elements, and scandium, titanium and tungsten of which possible functions are not found yet. The responses of trace elements to the exercise showed us that the selection and application of the convenient sport are important not only in terms of sportsman performance but also in terms of future healthy life plans and clinically. According to available literature, our study of these elements has not been studied previously in terms of exercise is important.
